# Serum Gastrin and Pepsinogen Levels after Administration of Acid Secretion Inhibitors for Ulcers due to Endoscopic Submucosal Dissection in Patients with Early Gastric Cancer

**DOI:** 10.1155/2022/2830227

**Published:** 2022-01-28

**Authors:** Maidina Abuduwaili, Tomoyuki Boda, Masanori Ito, Hidehiko Takigawa, Takahiro Kotachi, Taiji Matsuo, Shiro Oka, Shinji Tanaka

**Affiliations:** ^1^Department of Endoscopy, Hiroshima University Hospital, Hiroshima, Japan; ^2^Department of General Internal Medicine, Hiroshima University Hospital, Hiroshima, Japan; ^3^Department of Gastroenterology and Metabolism, Hiroshima University, Hiroshima, Japan

## Abstract

Acid secretion inhibitors, such as proton pump inhibitors (PPIs) and potassium competitive acid blockers (PCABs), are used to treat ulcers after endoscopic submucosal dissection (ESD) for early gastric cancer. These drugs can influence serum gastrin and pepsinogen (PG) levels; however, their definite effects remain unclear. This open-label, randomized study investigated the effect of acid secretion inhibitors on the serum gastrin and pepsinogen levels. In total, 76 patients were enrolled in the study. They underwent gastric ESD and received a PPI (*n* = 21) or PCAB (*n* = 55). Changes in the serum gastrin and PG levels before and 4 weeks after administration were examined. Patient factors associated with the alteration of serum PG or gastrin levels were identified. The median serum levels of gastrin, PGI, and PGII before the administration of the acid secretion inhibitors were 110.5 pg/mL, 36.4 ng/mL, and 8.9 ng/mL, respectively; after administration, the levels increased to 300 pg/mL, 64.7 ng/mL, and 15.8 ng/mL, respectively (*P* < 0.01). Univariate analysis revealed that PCABs led to a more significant increase in the serum gastrin and PG levels as compared to PPIs. Furthermore, the PG levels were significantly increased in patients with previous *Helicobacter pylori* infections than in those with current infections. In conclusion, the serum gastrin and PG levels increased after the use of acid secretion inhibitors. This elevation was affected by the type of drug used, whereas the elevation in PGs was affected by the patient's background as well.

## 1. Introduction

Endoscopic submucosal dissection (ESD) for gastric cancer is a minimally invasive and highly curative treatment that has gained popularity globally. However, bleeding from stomach ulcers (artificial ulcers) created following excision of the lesion has been a complication linked with ESD [1]. Thus, early healing of artificial ulcers is an urgent concern. For the treatment of ESD-induced stomach ulcers, proton pump inhibitors (PPIs) have been routinely employed [2, 3]. Recently, a new potassium competitive acid blocker (PCAB), vonoprazan, was reported to have a higher treatment potential for acid-related diseases (including artificial ulcers after ESD) than conventional agents [4]. In Japan, PPIs and PCABs have been shown to be the preferred drugs in the treatment of the increasing number of patients with gastroesophageal reflux disease [5, 6].

Gastrin, especially amidated gastrin-17 (G17), is only secreted by G cells and is the main circulating form in most mammals. Gastrin and pepsinogen (PG) reflect the function and state of the gastric mucosa and are considered representative biomarkers of the gastric physiology [7]. The use of PPIs/PCABs to suppress acid secretion may cause an increase in the serum gastrin and PG levels [8]. Thus, PPIs/PCABs stimulate the production of gastrin, which is also an effective growth factor; hypergastrinemia may induce hyperplasia of the enterochromaffin-like cells and may equally increase the risk of cell proliferation [9, 10]. We have previously reported that the PGI/PGII ratio and serum gastrin level are associated with the *Helicobacter pylori* (*H. pylori*) infection status [11] and that the risk of gastric cancer can be determined by measuring the PGI level and the PGI/II ratio [12]. As per acid inhibitory action, compared with PPI, PCAB may induce higher changes in gastrin and PG levels. Moreover, other factors, such as the state of *H. pylori*, may impact their blood levels. Therefore, the increased PG level after consumption of acid secretion inhibitors may not allow an effective histological evaluation of the gastric mucosa, thereby hindering diagnosis [13].

Acid secretion inhibitors are widely used; some patients consume them for a long time before the disease is cured. Thus, the influence of gastric acid inhibitors on blood gastrin and PG and the correlation between them should be considered [14]. In this study, we have investigated the increase in the serum gastrin and PG levels after PPI or PCAB administration and the factors related to the increase. Furthermore, we have examined the alteration in the serum gastrin fraction (G17/total gastrin).

## 2. Materials and Methods

### 2.1. Patients

In this retrospective cohort study, we enrolled 167 patients who underwent blood tests before and after ESD for gastric cancer between July 2017 and March 2019 at the Hiroshima University Hospital. Patients were excluded for PPI or PCAB usage before ESD; the use of anticholinergic drugs, antigastrin drugs, or histamine receptor-2 antagonists during the observation period; having the Zollinger–Ellison syndrome; having a history of gastrointestinal resection or vagus nerve ablation; and having autoimmune gastritis. Patients without an *H. pylori* infection were excluded: these are patients with no endoscopic gastric atrophy, no histologic atrophy of the gastric gland, no histologic inflammation of the gastric mucosa, no histologic *H. pylori* infection, and negative anti-*H. pylori* antibody titers.

To investigate the factors associated with the elevation of serum gastrin and PG levels after PPI or PCAB administration, the median amount of change in gastrin and PG levels was used to classify the group as large if it was higher than the median value and small if it was lower. The age, sex, drug type, degree of endoscopic atrophy (Kimura–Takemoto classification), and *H. pylori* infection status were compared between the two groups. Posteradication and naturally eradicated cases were designated as previous infection. Patients who were positive for endoscopic gastric atrophy and negative for HP antibody cases were classified as previous infection patients, whereas patients who were positive for both endoscopic gastric atrophy and HP antibody cases were classified as current infection patients. This study was registered as a retrospective cohort study (UMIN ID: UMIN000030407) and approved by the ethics committee of Hiroshima University (e-30518).

### 2.2. Estimation of Serum Markers

To fully evaluate the degree of changes in gastrin and pepsinogen caused by acid inhibitors, the values of these serum markers before and after administration were compared. Additionally, to examine the changes in G7 levels before and after administration, 10 cases from each group, the PPI group and PCAB group, were randomly selected for analysis. Fasting sera were collected on the day of the ESD and 4 weeks later and stored at -20°C until analysis. The levels of serum gastrin (Gastrin RIA Kit II; Dainabot, Tokyo, Japan), G17 (Gastrin-17 Advanced ELISA, Biohit, Finland), and PG (LZ test; Eiken, Tokyo, Japan) and the anti-*H. pylori* antibody titers (E-plate; Eiken, Tokyo, Japan) were evaluated [15].

### 2.3. Statistical Analysis

To compare the numerical data before and after medication, we used the Wilcoxon signed-rank test. We used the *χ*^2^ test and Fisher's exact test to analyze categorical data, as appropriate. Multivariate logistic regression analyses were performed to assess the factors associated with increased serum gastrin and PG levels. Confidence intervals were computed using normal approximation of the binomial distribution. *P* < 0.05 was considered statistically significant for each test. The JMP statistical software (SAS Institute Inc., Cary, NC, USA) was used for all statistical analyses.

## 3. Results

### 3.1. Changes in the Serum Gastrin, PGI, and PGII Levels after Administration of Acid-Suppressing Medications

**Figure 1:**
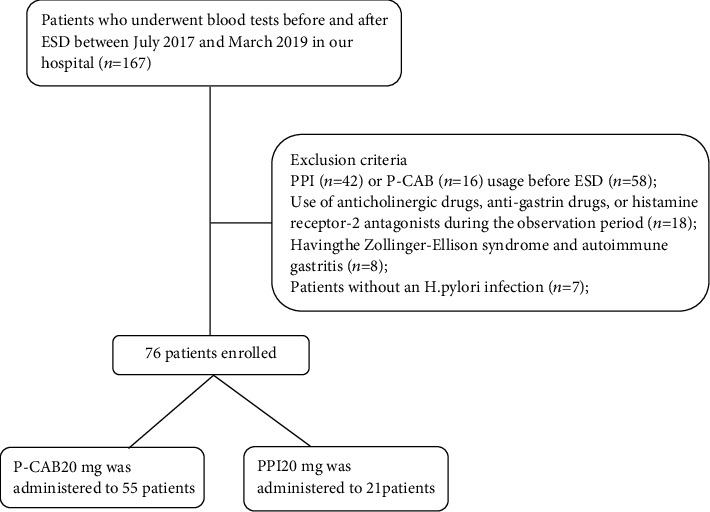
Flowchart showing data of patients enrolled in this study.

Finally, as shown in Figure 1, 76 patients (61 males; mean age, 69 years) were included in the analysis. Vonoprazan (PCAB; 20 mg) was administered to 55 patients who underwent ESD on either Monday or Wednesday, whereas esomeprazole (PPI; 20 mg) was administered to 21 patients who underwent ESD on the remaining weekdays.

**Figure 2:**
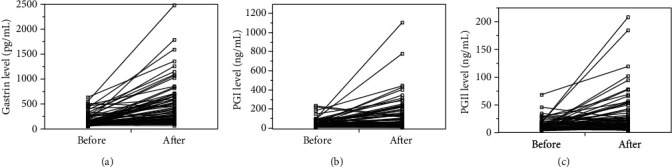
Serum levels of gastrin (a), pepsinogen I (PGI) (b), and pepsinogen II (PGII) (c) before and after administration of a proton pump inhibitor or potassium competitive acid blocker.

No significant differences were noted in age, sex, degree of endoscopic atrophy (Kimura–Takemoto classification), *H. pylori* infection status, and gastrin, PGI, and PGII levels before administration among the therapy groups (Table 1). After treatment with the PPI and PCAB, the gastrin, PGI, and PGII levels increased (Figure 2). Before the administration of acid secretion inhibitors, the median levels of serum gastrin, PGI, and PGII were 110.5 pg/mL, 36.4 ng/mL, and 8.9 ng/mL, respectively (Table 2). After administration, the levels significantly increased to 300 (a 167.5 pg/mL increase) pg/mL, 64.7 (a 24.5 ng/mL increase) ng/mL, and 15.8 (a 5.05 ng/mL increase) ng/mL, respectively.

### 3.2. Comparison of the Serum G17 Levels before and after PPI/PCAB Administration

**Figure 3:**
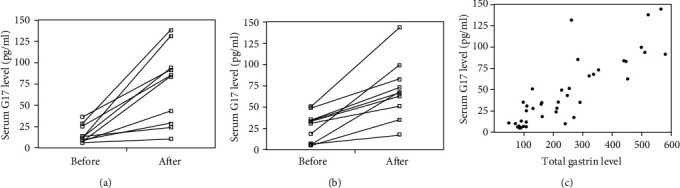
Comparison of serum G17 levels before and after proton pump inhibitor (a) and potassium competitive acid blocker administration (b). Correlation between serum gastrin level and G17 level (c).

Then, we examined the alteration in G17, the most significant subfraction of human gastrin. The serum G17 levels in patients treated with acid secretion inhibitors are presented in Figures 3(a) and 3(b). The serum G17 level increased significantly (Wilcoxon's signed-rank test *P* < 0.001) after administration of the PPI and PCAB.  Figure 3(c) shows the correlation between the G17 values and total gastrin values before and after administration in 20 patients; analysis revealed that the correlation coefficient between the two variables was 0.85, indicating a relatively strong correlation.

### 3.3. Host Factors for the Elevation of Serum Gastrin, PGI, and PGII Levels after Administration of Acid-Suppressing Medications

The large and small groups were then compared to examine the host factors that contributed to the differences in the extent of changes in the gastrin and PG levels. In the case of gastrin (Table 3), 89% of the patients in the large group used PCAB; the PCAB usage was significantly higher in the large group than in the small group (*P* = 0.001). Univariate analysis revealed that the drug type was the only host factor that contributed to an increased level of gastrin.

Conversely, in the case of PGs (Tables 4 and 5), statistically significant differences were observed in the drug type and *H. pylori* infection status between the two groups. Moreover, univariate analysis revealed significant intergroup differences in these two factors.

## 4. Discussion

In this study, we found that PPI or PCAB usage for ulcers caused by ESD increased serum gastrin and PG levels. These findings are consistent with the findings of several previous clinical trials and control studies [7, 16, 17]. Shiotani et al. [18] observed that the median serum gastrin level was higher in PPI users than in nonusers (234 vs. 113 pg/mL, *P* < 0.001) and higher in women than in men. Although there was no observable difference between men and women in our study, the levels tended to be higher in women, similar to that in Shiotani et al.'s report. It may be related to the difference in the number of research participants and period of administration.

Various adverse effects caused by the long-term use of PPI or PCAB are considered to be attributed to the characteristics of the drug and the subsequent biological changes. Hypergastrinemia may result from a long-term use of acid inhibitors in patients with chronic atrophic gastritis [19]. In animal models, hypergastrinemia caused by PPI reportedly increased the proliferation of adenomatous cells, and its association with increased gastric carcinoids has been confirmed [20, 21]. However, in our study, the median serum gastrin level following the administration of vonoprazan (20 mg) for 4 weeks was 2,470 pg/mL, which is similar to the level in a previous report by Iwakiri et al. [22]. Thus, patients may experience a sharp increase in the serum gastrin levels after the administration of acid secretion inhibitors. The association between long-term PPI/PCAB medication and hypergastrinemia-related disease is a subject for further research.

In recent years, Western countries have demonstrated that the serum level of G17, which is secreted from the antral G cells, has been used to evaluate the topography of gastritis by GastroPanel [23–26]. However, in Japan, the serum titer of gastrin has been used for the evaluation of corpus atrophy, and total gastrin is evaluated using the domestic enzyme-linked immunosorbent assay system. To the best of our knowledge, this study is the first to compare gastrin values within the same sample. An examination of the total gastrin and serum G17 levels revealed a close correlation between the two (*r* = 0.85, *P* < 0.001), as shown in Figure 2(c), and the G17 level was approximately 20% of the total gastrin fraction.

Factors that lead to increased serum levels of gastrin and PG should be investigated among PPI or PCAB users. Gastrin and its derivatives have long been considered cell growth factors and have been reported to have regulatory effects (such as antiapoptotic and proliferative effects) on some cells (including the gastric epithelium and cancer cells). Furthermore, some cytokines that induce G cells to secrete gastrin, such as ammonia and tumor necrosis factor-alpha, are released in *H. pylori*-associated gastritis, which further upregulates the gastrin levels [27]. In this study, our analysis revealed that the difference in the medications used (PPI or PCAB) was the main factor associated with the fluctuations in the gastrin levels. PCABs are novel acid-secreting inhibitors that have been shown to be more potent than PPIs in reducing gastric acid secretion [4]. The increase in gastrin level is caused by the increase in pH due to the suppression of acid secretion as a signal and the increase in gastrin secretion from G cells by feedback. Therefore, the increase in gastrin level depends on the strength of acid secretion suppression. We determined that PCABs induced more increases in gastrin levels than PPIs, which may be due to difference in their ability to inhibit gastric acid secretion.

The presence of *H. pylori*, severity of gastric atrophy, and inflammation may be the determinants of the serum PG levels. The reasons for the increase in PGs in PPI/PCAB users are unclear; however, possible causes may include hypertrophy or reproduction of the gastric acid glands and pyloric glands caused by high circulating gastrin and/or gastrin, which promote the release of PG in the gastric mucosa [10, 11, 25]. In line with these hypotheses, the serum PG levels in the PPI/PCAB users increased in our study. We speculated whether PPI/PCAB stimulated PG secretion in the gastric mucosa directly or through the gastrin connection. The serum PG levels tended to increase in patients with high gastrin levels, supporting the correlation between the PG and gastrin levels. In addition, we observed that both the elimination of inflammation after *H. pylori* eradication and less gastric atrophy led to an increase in the PG levels. In patients with moderate-to-severe atrophic gastritis, the average PG level was 16.3 ng/mL before eradication; this increased to 25.7 ng/mL after eradication [28]. Kishikawa et al. [29] reported that after eradication, the serum median PGI level significantly decreased from 51.9 ng/mL (29.7–68.6 ng/mL) to 30.1 ng/mL (24.3–40.3 ng/mL); the change of PGI by eradication therapy has been controversial. In our study, the change of PG level by responding to PPI/PCAB administration is larger in previous infection cases than in current infection cases, whereas the change of gastrin level by PPI/PCAB administration was not different. The responsiveness of PGs to PPI/PCAB treatment may be altered by eradication therapy.

This study has a few limitations. First, the sample size was relatively small, especially in PPI cases, since the patients who had taken PPI or PCAB before ESD were excluded from this study. There were more PPI users than PCAB users among the patients. Thus, the PPI group became smaller. Second, our research is a short-term study. Thus, the effect of long-term medication on serum gastrin and pepsinogen levels and the results need to be further confirmed in the future. Third, we targeted ESD patients as study subjects since we intended to assess how gastrin and PG levels changed in those who were at high risk of developing gastric cancer.

## 5. Conclusions

In summary, this study was conducted in a group of cases with high potential of carcinogenesis that developed to gastric cancer. In such population, PCAB resulted in a rapid increase in gastrin compared to PPI. This point should be considered when selecting PPI and PCAB; however, further consideration is required for its long-term effects.

## Figures and Tables

**Table 1:** Clinical characteristics of patients in each administration.

Characteristics	PPI (*n* = 21)	PCAB (*n* = 55)	*P* value
Age			
≤65	13 (61.9)	41 (74.5)	0.396
>65	8 (38.1)	14 (25.5)	
Gender			
M	16 (76.2)	45 (81.8)	0.748
F	5 (23.8)	10 (18.2)	
Endoscopic atrophy			
Close	4 (19.1)	4 (7.3)	0.159
Open	17 (80.9)	51 (92.7)	
HP infection status			
Present	4 (19.1)	20 (36.4)	0.177
Previous	17 (80.9)	35 (63.6)	
Gastrin^∗^	91 ± 110.8	130.1 ± 140.1	0.264
PGI^∗^	46.1 ± 31.5	47.4 ± 45.9	0.646
PGII^∗^	10.7 ± 6.6	12.9 ± 11.3	0.557

**Table 2:** Comparison of gastrin, PGI, and PGII levels before and after proton pump inhibitor/potassium competitive acid blocker administration.

	Before	After	*P* value	Amount of change
Gastrin (pg/mL)	110.5 (47–626)	300 (57–2470)	<0.0001^∗^	167.5 (-93–1930)
PGI (ng/mL)	36.4 (5.4–230.2)	64.7 (5.4–1098)	<0.0001^∗^	24.5 (-106.6–1003.5)
PGII (ng/mL)	8.85 (2.6–67.8)	15.8 (3.6–207)	<0.0001^∗^	5.05 (-21.1–190.4)

**Table 3:** Univariate analysis of the host factors that contribute to serum gastrin level alterations.

Variables *n* (%)	Change of gastrin value		Univariate OR (95% CI)	*P* value
	Large (*n* = 38)	Small (*n* = 38)		
Age (years)				
≥65	30 (55.6)	24 (44.4)	1	
<65	8 (36.4)	14 (63.6)	0.46 (0.16–1.27)	0.129
Sex				
Male	29 (47.5)	32 (52.5)	1	
Female	9 (60)	6 (40)	1.66 (0.52–5.22)	0.565
Medicine				
PPI	4 (19)	17 (81)	1	
PCAB	34 (61.8)	21 (38.2)	6.88 (2.04–23.2)	0.001^∗^
Endoscopic atrophy				
Closed	4 (50)	4 (50)	1	
Open	34 (50)	34 (50)	1 (0.23–4.33)	1.0
HP infection status				
Present	12 (50)	12 (50)	1	
Previous	26 (50)	26 (50)	1 (0.38–2.63)	1.0

∗ indicates statistically significant values. A median value ≥ 167.5 is large.

**Table 4:** Univariate analysis of the host factors that contribute to serum PGI level alterations.

Variables *n* (%)	Change of PGI value		Univariate OR (95% CI)	*P* value
	Large (*n* = 38)	Small (*n* = 38)		
Age (years)				
≥65	25 (46.3)	29 (53.7)	1	
<65	13 (59.1)	9 (40.9)	1.67 (0.61–4.57)	0.312
Sex				
Male	31 (50.8)	30 (49.2)	1	
Female	7 (46.7)	8 (53.3)	0.85 (0.27–2.62)	1.0
Medicine				
PPI	6 (28.6)	15 (71.4)	1	
PCAB	32 (58.2)	23 (41.8)	3.48 (1.17-10.30)	0.021^∗^
Endoscopic atrophy				
Closed	7 (87.5)	1 (12.5)	1	
Open	31 (45.6)	37 (54.4)	0.12 (0.01–1.03)	0.056
HP infection status				
Present	7 (29.2)	17 (70.8)	1	
Previous	31 (59.6)	21 (40.4)	3.59 (1.27–10.12)	0.014^∗^

∗ indicates statistically significant values. A median value ≥ 24.45 is large.

**Table 5:** Univariate analysis of the host factors that contribute to PGII level alterations.

Variables *n* (%)	Change of PGII value		Univariate OR (95% CI)	*P* value
	Large (*n* = 38)	Small (*n* = 38)		
Age (years)				
≥65	25 (46.3)	29 (53.7)	1	
<65	13 (59.1)	9 (40.9)	1.66 (0.61–4.57)	0.312
Sex				
Male	31 (50.8)	30 (49.2)	1	
Female	7 (46.7)	8 (53.3)	0.85 (0.27–2.62)	1.0
Medicine				
PPI	6 (28.6)	15 (71.4)	1	
PCAB	32 (58.2)	23 (41.8)	3.48 (1.17–10.30)	0.021^∗^
Endoscopic atrophy				
Closed	7 (87.5)	1 (12.5)	1	
Open	31 (45.6)	37 (54.4)	0.12 (0.01–1.03)	0.056
HP infection status				
Present	6 (25)	18 (75)	1	
Previous	32 (61.5)	20 (38.5)	4.8 (1.63–14.1)	0.003^∗^

∗ indicates statistically significant values. A median value ≥ 5.05 is large.

## Data Availability

The data that support the findings of this study are available from the corresponding author upon reasonable request.
